# Supplementary material for: Biomarkers to predict or measure steroid resistance in idiopathic nephrotic syndrome: A systematic review
Source: PLoS One. 2025 Feb 13;20(2):e0312232. doi: 10.1371/journal.pone.0312232 (PMC11824968; doi:10.1371/journal.pone.0312232)
Supplement: Attachment — Submitted filename: Systematic Review PLOS ONE Reviewers Rebuttal.docx [file pone.0312232.s003.docx]

**Reviewer #1 Comments**

TITLE – The title denotes predictive markers but the data presented shows both predictive and evaluative markers… please edit title to more accurately reflect the data you are presenting.

- **I have amended the title from “Biomarkers to predict steroid resistance in idiopathic nephrotic syndrome: a systematic review.” to “Biomarkers to predict or measure steroid resistance in idiopathic nephrotic syndrome: a systematic review.”.**

Figure 2 - Reference to Figure 1 here seems incorrect.

- **I erroneously referred to figure 2 instead of figure 1. This has now been amended**

Please add details about the BIOCROSS methodology and scoring metrics to improve readability.

- **Thank you for pointing this out, we have added the following paragraph to the methods section: “**BIOCROSS is a quality assessment tool that is used to quantify the quality of the data that supports candidate biomarkers. The methodology of this tool is covered including details of the scoring is covered here . Briefly, the tool includes 10-items covering 5 domains: ‘Study rational’, ‘Design/Methods’, ‘Data analysis’, ‘Data interpretation’ and ‘Biomarker measurement’, aiming to assess different quality features of biomarker cross-sectional studies.”

Figure 3 – Please add details about this assessment here as well.

- **Again, thanks for pointing this out, we have added the following: “The QUADAS-2 tool assesses the design and publication of biomarker data for applicability and risk of bias across four domains: patient selection, index test, reference standard, and flow and timing. It allows researchers to compare the risk of bias across different studies. The specific methodology is covered in detail here.”**

**Details of the domains and their respective signalling questions have been added to the manuscript as follows:**

**“DOMAIN 1: PATIENT SELECTION Risk of bias: Could the selection of patients have introduced bias?**

Signalling question 1: Was a consecutive or random sample of patients enrolled? Signalling question 2: Was a case-control design avoided? Signalling question 3: Did the study avoid inappropriate exclusions?

**DOMAIN 2: INDEX TEST Risk of Bias: Could the conduct or interpretation of the index test have introduced bias?**

Signalling question 1: Were the index test results interpreted without knowledge of the results of the reference standard? Signalling question 2: If a threshold was used, was it pre-specified?

**DOMAIN 3: REFERENCE STANDARD Risk of Bias: Could the reference standard, its conduct, or its interpretation have introduced bias?**

Signalling question 1: Is the reference standard likely to correctly classify the target condition? Signalling question 2: Were the reference standard results interpreted without knowledge of the results of the index test?

**DOMAIN 4: FLOW AND TIMING Risk of Bias: Could the patient flow have introduced bias?**

Signalling question 1: Was there an appropriate interval between index test and reference standard? Signalling question 2: Did all patients receive the same reference standard?”

Figure 4 – Please add details here as well… The readers cannot be expected to be familiar with your methods…

- **Figure 4 shows a plot of sensitivity vs specificity, We feel the figure legend is sufficient as this is a simple synthesis. “Each biomarker or panel had its specificity plotted against its sensitivity. Points were awarded according to the increasing level of specificity and sensitivity.”**

Please either list each marker in rank order in the text based on your criteria for usefulness, or at least group them into 2-4 tiers in order of strength of evidence to help the readers understand the final results of your detailed analyses.

- **We appreciate your point of view. The order of the biomarkers presented in figures 2 and 5 is the same as the order that they were presented following the original literature search. We have added a sentence to the figure legends of figure 2 and figure 5 to make this clear, “candidate biomarkers are presented in the same order as revealed by the literature search”. We agree that 2-4 tiers in order of strength of evidence may well help the reader. However, the boundaries of these tiers would be somewhat arbitrary. As such we may unduly influence the reader as to the validity of each biomarker. As the review stands we are laying the evidence bare and are objectively scoring the biomarkers and their respective source manuscripts based on published quality assessment tools. Respectfully, we have left the tables presented in figures 2 and 5 as they are in terms of the order of the presented biomarkers.**

Please clearly classify all markers throughout the text as either serum, plasma, or urine markers. Indeed, the manuscript could arguably be more useful to readers if it were organized by serum vs. plasma vs. urine biomarkers.

- **There is a column in Figure 2 that indicates the source of the biomarker, for increased clarity this column has been added to the table shown in Figure 5. We feel that subdividing the biomarkers based on their source would make comparing the relative performance of each candidate more difficult**

VDBP paragraphs appear twice and should be merged…

- **Thank you, these have been merged.**

Some markers discussed (Fetuin-A, AGP, etc…) do not seem to even be on your list of 17 in Figure 2. Please correct and/or reconcile this.

- **Fetuin-A and AGP are present in the panels of biomarkers. We have now made sure that all biomarkers from both panels are covered. Additionally we have made it clear in the prose that the some of the biomarkers are effective as part of a panel rather than in their own right.**

AGP-1 needs to be defined.
MRP-1 needs to be defined.

- **These definitions have now been added.**

Some references appear to be incorrect. Please verify the accuracy of all references throughout the manuscript.

- **Thanks for pointing this out. We have now manually verified ech reference individually and are satisfied that they are accurate.**

Figure 3 – Please add a column at left side to name each biomarker or panel to enhance readability of the table. Also, the text below “Reference Standard” seems incorrect and needs editing.

- **Adding the full name of each biomarker would make the tables cumbersome and could well reduce the readability of the tables. This information has been included elsewhere and is possibly tautologous to include here too. However, we totally agree that naming the biomarkers that comprise the panel’s would be useful here and so we have added this to Figure 2 and Figure 5**

Figure 4 – This figure nicely separates the markers by quality of evidence but is uninterpretable without the addition of the actual names of the markers on the figure… Please add individual names instead of numbers that require the reader to find another table where they were numbered. Also, please change both axes to stop at 100%. A potential alternative would be to create a table of the biomarkers that is organized by quartiles based on the strength of evidence.

- **We appreciate and understand your input. We have kept the numbers in the graph to stop it becoming too confusing, but have added a key so that the numbers can be more easily interpreted without searching through the manuscript. We have limited each axis to 100%**

Figure 5 – Please consider separating the markers in this table based on predictive vs. evaluative, and list them in order of strength of evidence to enhance the interpretability for the readers.

- **Thank you for pointing this out. We have rearranged the layout of the table shown in Figure 5 to show predictive and evaluative markers listed in order of strength of evidence.**

**Reviewer #2 Comments**

The function of each biomarker on the kidney is described at length in the results section. Finally, suPAR and haptoglobin were selected as the most promising biomarkers for the distinction between SSNS and SRNS. However, in the discussion section, their importance is not highlighted. It is inappropriate.

- **We apologise for this and have now added some information towards the end of the discussion section.**

There are some linguistic gleanings that require a careful revision. The paper needs to be edit with the English editing companies.

- **This was a criticism not identified by the other reviewers, nevertheless I have enlisted the help of a couple of native English-speakers (like me) to proof-read the manuscript.**

the paper is not well-written, figures are vague and hardly to read.

- **I am at a bit of a loss as to how to address this criticism save for re-reading the manuscript with this viewpoint in mind. Hopefully by addressing all the comments described by yourself and the other reviewers you will find the revised manuscript much improved.**

**Reviewer #3 Comments**

This review is mainly a list of biomolecules; it is not a negative point, but often it lacks information, and it is not clear how the molecules are listed. I suggest to the authors to separate the biomarkers into groups like useful as early treatment, useful prior to treatment, and not useful (like AGP1, which seems able only to identify INS).

- **Thank you for your comment. Indeed, reviewer 1 identified a similar issue. We have now made clear that in Figure 2 the biomarkers are listed in the order they appeared when we performed the literature search, and in Figure 5 the biomarkers are now listed by category in terms of whether they are predictive or evaluative. I am not sure I understand your point regarding AGP-1. AGP-1 seems to be a marker of kidney lesions and has been found to be elevated in a range of kidney diseases. It is included in this systematic review as part of the panel of 10 biomarkers published by Bennet *et al* in 2017**

The title of each molecule could be uniform. Actually, some of the themes are presented as acronyms and others as full names. I suggest writing with your full name and acronym.

- **You are correct. We have now amended the manuscript in response to this recommendation. The tables in Figures 2 and 5 now have the name of the biomarker and, where appropriate, the acronym. In the prose, each biomarker is introduced by its full name and then the acronym underneath. We hope this increases clarity for the reader.**

VDBP is presented twice; please fuse them into one paragraph.

- **This has now been rectified.**

It is important for each molecule to report the obtained results from considered studies and the obtained score at your analysis.

- **The scores calculated and shown in Figure 5 are based on each candidate biomarker or panel of biomarkers. The scores are laid out in separate columns along with a sum of the scores.**

As the authors wrote regarding MDR1’s SNP, it could also be useful to write about other possible SNPs able to distinguish SR from no responder.

- **We totally agree. However, due to the way systematic reviews are performed we are only able to include manuscripts that were identified following the literature search and according to the inclusion and exclusion criteria. We will keep this in mind for future work as genotyping tools would be very valuable.**
